# Learning by Exposure in the Visual System

**DOI:** 10.3390/brainsci12040508

**Published:** 2022-04-17

**Authors:** Bogdan F. Iliescu, Bryan Hansen, Valentin Dragoi

**Affiliations:** 1 Neurosurgery Department, Gr T Popa University of medicine and Pharmacy, 700115 Iasi, Romania; 2 Neurobiology and Anatomy, University of Texas-Houston Medical School, Houston, TX 77030, USA; bryan.hansen@tmc.uth.edu (B.H.); valentin.dragoi@tmc.uth.edu (V.D.)

**Keywords:** exposure, plasticity, visual system, perceptual learning

## Abstract

It is increasingly being understood that perceptual learning involves different types of plasticity. Thus, whereas the practice-based improvement in the ability to perform specific tasks is believed to rely on top-down plasticity, the capacity of sensory systems to passively adapt to the stimuli they are exposed to is believed to rely on bottom-up plasticity. However, top-down and bottom-up plasticity have never been investigated concurrently, and hence their relationship is not well understood. To examine whether passive exposure influences perceptual performance, we asked subjects to test their orientation discrimination performance around and orthogonal to the exposed orientation axes, at an exposed and an unexposed location while oriented sine-wave gratings were presented in a fixed position. Here we report that repetitive passive exposure to oriented sequences that are not linked to a specific task induces a persistent, bottom-up form of learning that is stronger than top-down practice learning and generalizes across complex stimulus dimensions. Importantly, orientation-specific exposure learning led to a robust improvement in the discrimination of complex stimuli (shapes and natural scenes). Our results indicate that long-term sensory adaptation by passive exposure should be viewed as a form of perceptual learning that is complementary to practice learning in that it reduces constraints on speed and generalization.

## 1. Introduction

It is generally accepted that exposure to incoming stimuli during perception induces plasticity in a way that modifies sensory representations. Perceptual plasticity can have different manifestations defined by their behavioral characteristics and the way in which they are induced. The major form of perceptual plasticity is perceptual learning, which is the practice-induced improvement in the ability of a sensory system to perform specific tasks. It is believed that perceptual learning occurs when stimuli are linked to a specific task that requires attention and is practiced repeatedly, possibly by increasing attention to important stimuli and decreasing attention to irrelevant stimuli [1,2,3,4,5]. Several lines of evidence indicate that practice-based perceptual learning is governed by top-down processes that cause long-term changes in the representation of attended stimuli and a corresponding improvement in behavioral performance [6,7,8].

In the visual system, practice learning is known to gradually improve the capacity of human subjects to detect small changes in a variety of simple stimuli, ranging from simple visual attributes, such as orientation, position, and motion [8,9,10,11,12,13,14], to complex stimuli, such as shapes and texture [15,16,17]. A key characteristic of this improvement is stimulus specificity. That is, the improvement in performance is typically restricted to stimuli that are similar to those present during training, with transfer to similar stimuli presented in a different context under certain conditions (appropriate tasks and parameters [9,10,11,13,14,18,19,20]. Perceptually, short-term exposure (seconds to minutes) to a fixed oriented grating has been shown to improve orientation discrimination performance around [21,22] and orthogonal ([21,23], for effects induced by brief adaptation) to the adapting orientation, and impair discrimination performance at intermediate orientations [21]. However, whether the orientation discrimination performance changes systematically across days of exposure has never been examined.

A second form of perceptual plasticity is adaptation. It differs from practice learning in that it is induced by passive stimulus exposure rather than by task repetition; hence, it is believed to rely on bottom-up plasticity. Adaptation is commonly viewed as a short-term phenomenon that reduces responses to frequent stimuli [23,24] and increases responses to infrequent stimuli [23,24,25] in a way that enhances the sensitivity to stimulus changes during perception. However, adaptation is believed to be unable to cause long-term changes in perceptual performance. This view has been challenged by several studies showing that repeated exposure, or adaptation, to a stimulus, even when it is behaviorally irrelevant or presented below the visibility threshold, is able to influence perceptual decisions [26,27] and induce learning [28]. This raises the possibility that perceptual performance can be improved not only through attention-based perceptual (or practice) learning, but also through repetitive exposure to behaviorally irrelevant, unattended stimuli (exposure learning). Since these two forms of learning clearly rely on different types of mechanisms (top-down and bottom-up), examining practice learning and exposure learning concurrently could lead to a better understanding of the way in which the brain adapts to different types of information to control behavior. However, despite the importance of this issue, the relationship between top-down and bottom-up perceptual plasticity is not well understood.

This issue has been difficult to address until now because of the lack of complete information regarding the capacity of sensory systems to adapt as a result of passive exposure (see Discussion). Here we examined the relationship between practice and exposure learning by investigating perceptual learning in the orientation domain, as orientation is a key local feature that is essential for defining complex shapes in natural scenes, and has a distinct representation in the visual cortex of higher mammals. First, we asked whether learning could arise through simple exposure to oriented stimuli. We found that whereas repetitive exposure to a fixed orientation did not increase or decrease orientation discrimination performance, daily exposure to orthogonal orientation sequences induced a spatially localized, orientation-specific improvement in orientation discrimination around the ‘exposed’ axes.

Second, we tested the relationship between exposure and practice-based orientation learning by focusing on two basic properties of learning, i.e., the rate of improvement in performance and the capacity for stimulus generalization. Contrary to expectation, we found that: (i) the improvement in performance following passive exposure to orientation sequences was stronger than the improvement in performance after subjects practiced orientation discriminations; and (ii) the orientation-specific improvement in orientation discrimination performance due to passive exposure led to a robust improvement in the discrimination of shapes and natural scenes with orientation components around the exposed axes, whereas perceptual orientation learning showed only weak transfer to complex stimuli. Altogether, these results indicate that long-term sensory adaptation by passive exposure should be viewed as a form of perceptual learning that is complementary to task-related learning in that it reduces constraints on speed and generalization.

## 2. Materials and Methods

### 2.1. Subjects

Six adult human subjects (3 males, 3 females, 24–40 years of age) with normal or corrected to normal vision participated in the experiments. Subjects were presented stimuli binocularly on a computer screen of uniform gray background (35 cd m^−2^) at a viewing distance of 55 cm. Each subject was naive with respect to the orientation discrimination task and participated in all the experiments.

### 2.2. Exposure Phase

Exposure sessions were conducted twice a day. Stimuli during the exposure phase consisted of high-contrast (80%), 2.2 cpd sinusoidal circular gratings (3° in diameter) presented at 5° eccentricity with respect to a central fixation point on a horizontal axis (size 0.1° and 100% contrast, that always remained on the center of the screen). The exposure phase consisted of 300 presentations of stimuli separated by blanks (each stimulus was flashed for 200 ms and separated from the next stimulus by a 200 ms blank interval; the exposure phase lasted 180 s). The orientation of the exposure stimuli was either fixed (60° or 150°) or variable (alternating 60°/30° and 60°/150° sequences presented in different sessions). 

To prevent interference between the effects induced by the different exposure stimuli that we tested, we used different, non-overlapping, spatial locations for each set of exposure stimuli. Throughout the exposure phase, attention was controlled by asking subjects to complete a letter identification task (presented 7° above the fixation point), in which a new letter was randomly drawn from a letter pool and presented every 200 ms (consecutive letters were separated by a 200 ms blank) at the same time that a grating was presented at the exposed location (Supplementary Information). Subjects were required to count how many times a specific letter in the sequence was presented while they maintained fixation and were stimulated at the exposed location (letters were presented at 100% contrast and were 3° in size). We presented this task only after subjects became familiar with letter identification and their performance in the task became stable (if the slope of the linear fit from the last 12 reversals was less than 0.02). The target letter was randomly selected from a set of eight different letters at the beginning of each exposure session. At the end of each session, subjects had to report the number of times a specific letter appeared in the sequence, and then their number was compared to the actual number registered by the computer. The exposure session was repeated if the performance in the letter identification task had an accuracy below 80%. The mean count performance across sessions (averaged across all six subjects) was approx. 90%.

### 2.3. Orientation Discrimination

**Figure 1:**
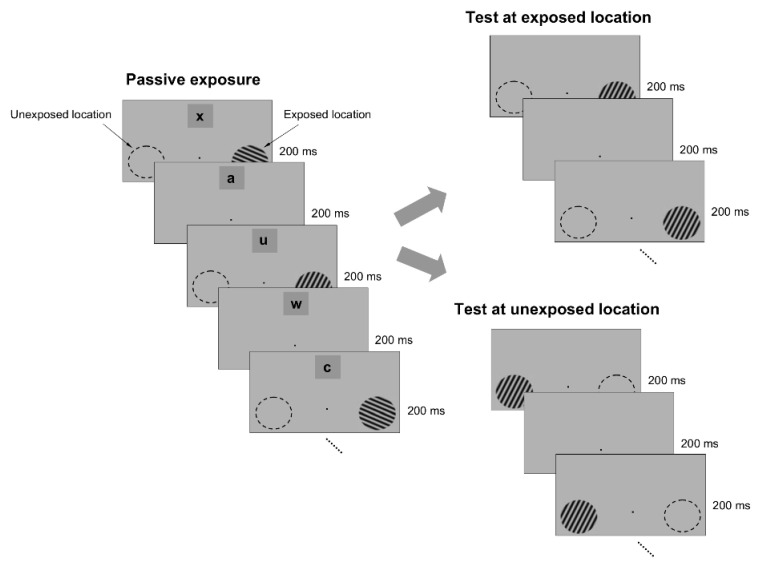
Exposure paradigm. The exposure phase consisted of successive presentations of specific orientations (3 ° circular sine-wave gratings) that were flashed for 200 ms each at an exposed location for several minutes. Exposure was performed only at the exposed location, while another, unexposed, location, was not stimulated. Throughout the exposure stage, a letter identification task (letters were located 7 ° away from the fixation point) was paired with the exposure stimuli (each letter was flashed for 200 ms). At the end of the exposure session, subjects were required to recall how many times a specific letter occurred in the sequence. The exposure stage was followed by a delayed match-to-sample orientation discrimination task around the experienced orientations, conducted both at the exposed and unexposed locations. One stimulus (target grating) had an orientation that always matched the orientation of the exposed stimuli, whereas the second stimulus (test grating) had a random orientation that was within 5° of the target. The target appeared first on 50% of the trials. At the end of each trial, subjects had one second to press a button whenever the target and test stimuli had a similar orientation.

**Figure 2:**
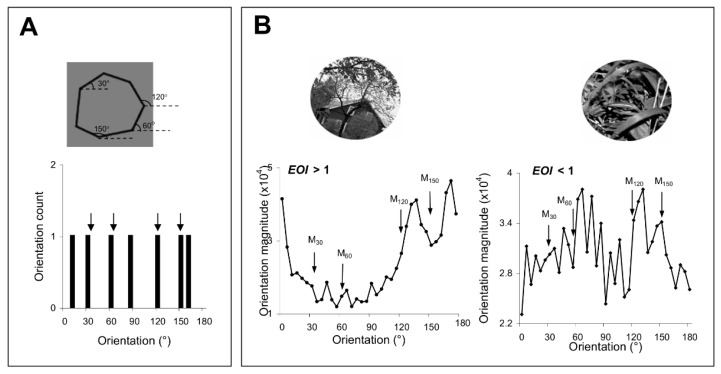
(**A**) Shape discrimination. We generated polygons with four of the sides oriented along the exposed axes, i.e., 30°, 60°, 120°, and 150°. The histogram below indicates the orientation distribution of the corresponding shape, with arrows pointing toward the orientations presented during the exposure stage. (**B**) Image discrimination. Two representative images and their corresponding exposure orientation index, EOI, which quantifies the relative strength of the orientation signals along exposed axes in each image. For each image, we determined the orientation at each pixel location based on the direction of the local gray-scale gradient in a standard 25-pixel array from the arc tangent of the partial derivative of brightness in a 5 × 5 kernel in the vertical direction, divided by this value in the horizontal direction [13]. The results for all the pixels are represented as orientation magnitude histograms (below each image). Arrows point toward the orientations presented during exposure.

At the end of the exposure phase, subjects completed orientation discrimination tasks at both the exposed and unexposed locations (these locations were symmetrical with respect to the fixation point). In this task, a target was flashed for 200 ms at an orientation that was identical to one of the exposed orientations (e.g., θ = 30°, 60°, or 150° for the experiments in Figure 1 and Figure 2). Following a delay of 200 ms, a test grating, which was randomly chosen from a set of test stimuli that differed by 0° or by ±1°, ±2°, ±3°, and ±4° with respect to the target orientation, was flashed for 200 ms. At the end of each trial, subjects were asked to press a button within a 1\s interval whenever the test orientation matched that of the target (there were 10 trials for each orientation difference between the target and test stimuli; the number of match and non-match stimuli was identical). The order between the target and test stimuli was random across trials (the target appeared first on 50% of the trials). Tests were performed at both the exposed and unexposed locations (in random order) at least 30 min after each exposure session. At the end of each discrimination session, we applied signal detection theory (cf. Green and Sweets, 1966) to calculate the orientation discrimination performance, d’, for each orientation difference using the equal-variance model (Supplementary Information).

### 2.4. Shape Discrimination

We constructed target shapes by creating heptagons with four of the sides oriented along the experienced axes, (i.e., 30°, 60°, 120°, and 150°, Figure 2A). Each shape was presented at an 80% contrast. The circle circumscribing the polygon had the same diameter as that of the exposure stimuli. We constructed test stimuli by rotating target stimuli by 0°, ±2°, ±3°, ±4°, and ±5° around their center of mass (using Adobe Photoshop 7.0^®^). In each trial the target was presented for 250 ms followed by a delay of 200 ms, and then a test stimulus was flashed for 250 ms. At the end of each trial, subjects were required to indicate if the target matched the test by pressing a keyboard button (each test stimulus was presented 10 times; the number of match and non-match stimuli was identical). We devised ‘control’ shapes by rotating the target and test polygons around their center of mass by 20° in a clockwise direction, and then repeated the shape discrimination task. Shape discrimination experiments were performed at both the exposed and unexposed locations. At the end of each discrimination session, we calculated the shape discrimination performance, d’, for each test stimulus using the equal-variance model.

### 2.5. Natural Images

We extracted 20 natural images from a high-resolution, commercial photo-CD library. All images were circularly clipped such that their diameter equaled the diameter of the exposure stimuli. To eliminate possible artifacts due to the use of images composed of one dominant orientation, we did not consider images that had a unimodal orientation distribution (Figure 2B). Images were transformed by eliminating color information, and then rotated by ±2°, ±3°, ±4°, and ±5° around their center of mass to generate test stimuli (using Adobe Photoshop 7.0^®^). In each trial a target image was flashed for 250 ms followed by a delay of 200 ms and a test image was flashed for 250 ms. At the end of each trial, subjects were required to indicate if the target and test images were identical by pressing the keyboard button (each test stimulus was presented 10 times; the number of match and non-match stimuli was identical). In control experiments, we rotated the target and test images by 20° in a clockwise direction to generate ‘rotated’ images. The experimental paradigm was similar to the shape discrimination paradigm, and was carried out at both the exposed and unexposed locations. At the end of each discrimination session, we calculated the image discrimination performance, d’, for each test stimulus using the equal-variance model.

For each image we calculated the orientation histogram 23, and then an exposure orientation index, EOI, was computed as follows:

$$\begin{array}{c} N\\ \sum \;\;M\theta i/Nexposedexposed\\ EOI\;=\;i\;=\;1\\ \sum \;\;M\theta j/NtotalNtotal\\ j\;\;=\;1 \end{array}$$

where Mθi represents the orientation magnitude obtained for the set of Nexposed = 4 orientations presented during exposure, θi; Mθj represents the orientation magnitude obtained for the set of Ntotal = 36 orientations, θj, j = 0, 1... N − 1, which are uniformly distributed over 0°–180°.

## 3. Results

### 3.1. Learning by Passive Exposure to Oriented Stimuli

To test whether passive exposure to oriented stimuli induces learning, we asked subjects to fixate on a small dot in the center of a computer screen while oriented sine-wave gratings were repeatedly flashed for 200 ms each, and separated by a 200 ms blank interval (Figure 1). The oriented stimuli were presented only at an ‘exposed’ location during a typical 3 min exposure session, while another location, symmetric with respect to the fixation point, was not stimulated (Figure 1). Throughout the exposure stage, we controlled attention by asking subjects to complete a letter identification task (presented 7° above the fixation point), in which a new letter was randomly presented every 200 ms (Figure 1). Subjects were required to count how many times a specific letter in the sequence was presented while they maintained fixation and were stimulated at the exposed location. We chose the location of the letter task based on pilot tests (Supplementary Figure S1), conducted prior to the exposure experiments, such as to ensure that the exposed and unexposed locations were unattended when subjects performed the letter task at a performance level of 80% or more.

To examine whether passive exposure influences perceptual performance, we asked subjects to test their orientation discrimination performance around and orthogonal to the exposed orientation axes, both at the exposed and unexposed locations (the discrimination tests were performed after the completion of each exposure session). We measured orientation discrimination performance using a delayed match-to-sample task in which subjects were required to report whether two circular gratings, briefly flashed for 200 ms, and separated by a 200 ms blank, differed in orientation (Figure 1). One of the gratings (target) had a fixed orientation, whereas the orientation of the other grating (test) was within 4° of the first (orientation was randomly varied in steps of ±1°). This allowed us to measure whether and how the orientation discrimination performance (quantified by the d’) changed at the exposed and unexposed locations after each exposure session.

**Figure 3:**
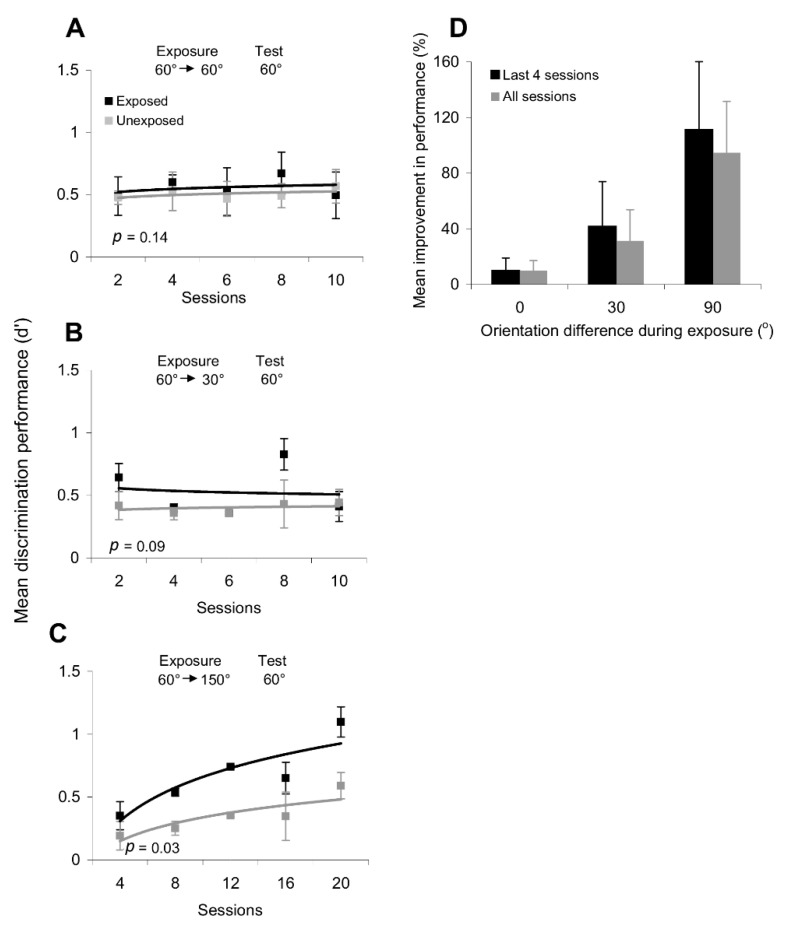
Changes in orientation discrimination performance (d’) after exposure to different orientation sequences. (**A**,**B**) Repetitive exposure to one orientation (60°) does not alter orientation discrimination performance around (**A**) and orthogonal (**B**) to the exposed orientation. (**C**) Exposure to non-orthogonal orientation sequences (60°—30°) does not lead to a significant change in the discrimination performance at the exposed location. (**D**) Exposure to orthogonal orientation sequences (60—150°) leads to a significant change in the discrimination performance around 60° at the exposed location. There was a similar improvement in discrimination performance around the orthogonal orientation (150°). This effect was measured over 20 exposure sessions and was statistically significant in each subject (*p* < 0.05, Student’s *t*-test). Black line and symbols represent the orientation discrimination performance (d’) at the exposed location; gray line and symbols represent the orientation discrimination performance (d’) at the unexposed location. Error bars represent SEM.

The effects of orientation exposure on the discrimination performance are summarized in Figure 3. We found that repetitive passive exposure to one orientation did not improve or impair orientation discrimination performance along parallel or orthogonal axes. Indeed, as shown in Figure 3A, after exposure to 60° orientations, there was no difference between the discrimination performance at the exposed and unexposed locations (*p* > 0.1, Student’s *t*-test). When the orientation discrimination performance was tested around the orthogonal orientation (Figure 3B), we found that exposure caused an early increase in discrimination performance at the exposed location (exposure to 60° and test at 150°), but performance in the subsequent sessions did not differ significantly from that at the unexposed location (*p* > 0.1, Student’s *t*-test).

We further tested the possibility that repetitive exposure to sequences of different orientations could induce systematic changes in discrimination performance. Thus, we repeatedly exposed subjects to sequences of non-orthogonal (60°/30°) and orthogonal orientations (60°/150°). However, whereas repeated exposure to the 60°/30° sequence led to a non-significant improvement in orientation discrimination performance around 60° (*p* = 0.09, Student’s *t*-test; Figure 3C), only exposure to an orthogonal orientation sequence led to a significant improvement in the discrimination of orientations along the exposed axes (for discrimination performance around 60°: *p* = 0.03, Student’s *t*-test; Figure 3D—similar results were obtained when we tested orientation discrimination around 150°).

The session-by-session improvement in orientation discrimination performance after passive exposure to a series of orthogonal gratings was observed only at the exposed location, despite the fact that subjects had similar training in discriminating stimuli around the experienced orientations at both the exposed and unexposed locations. Indeed, after only four exposure sessions (Figure 3D), subjects were able to correctly perceive increasingly smaller differences in orientation at the exposed location, which is equivalent to an increase in d’, whereas only a weak improvement in discrimination performance was observed at the unexposed location. These results are consistent with recent perceptual and physiological findings on orientation adaptation indicating that short-term adaptation to a grating of fixed orientation sharpens the orientation tuning curves of orthogonal V1 neurons and improves the perceptual discrimination performance around the orthogonal orientations [23,24,29], while broadening the tuning curves of the neurons tuned to all other orientations and impairing the discrimination of intermediate orientations [23,24,29]. This indicates that neuronal circuits in early and mid-level visual cortical areas are selectively altered by exposure to particular orientation sequences to modulate orientation discrimination performance in a way that induces learning.

The learning effects induced by repetitive exposure to orthogonal orientations are persistent. We found (data not shown) that, although the improvement in orientation discrimination performance gradually diminished when exposure was abolished, the discrimination performance remained significantly stronger at the exposed location even at 120 days after exposure was ceased (*p* < 0.05, Student’s *t*-test). These results are inconsistent with attentional learning theories [11,24,30], which postulate that only attended stimuli induce long-lasting changes in sensory systems, and suggest that learning can arise through purely bottom-up mechanisms. Thus, repeated training on a specific visual task is not a necessary prerequisite of perceptual learning.

**Figure 4:**
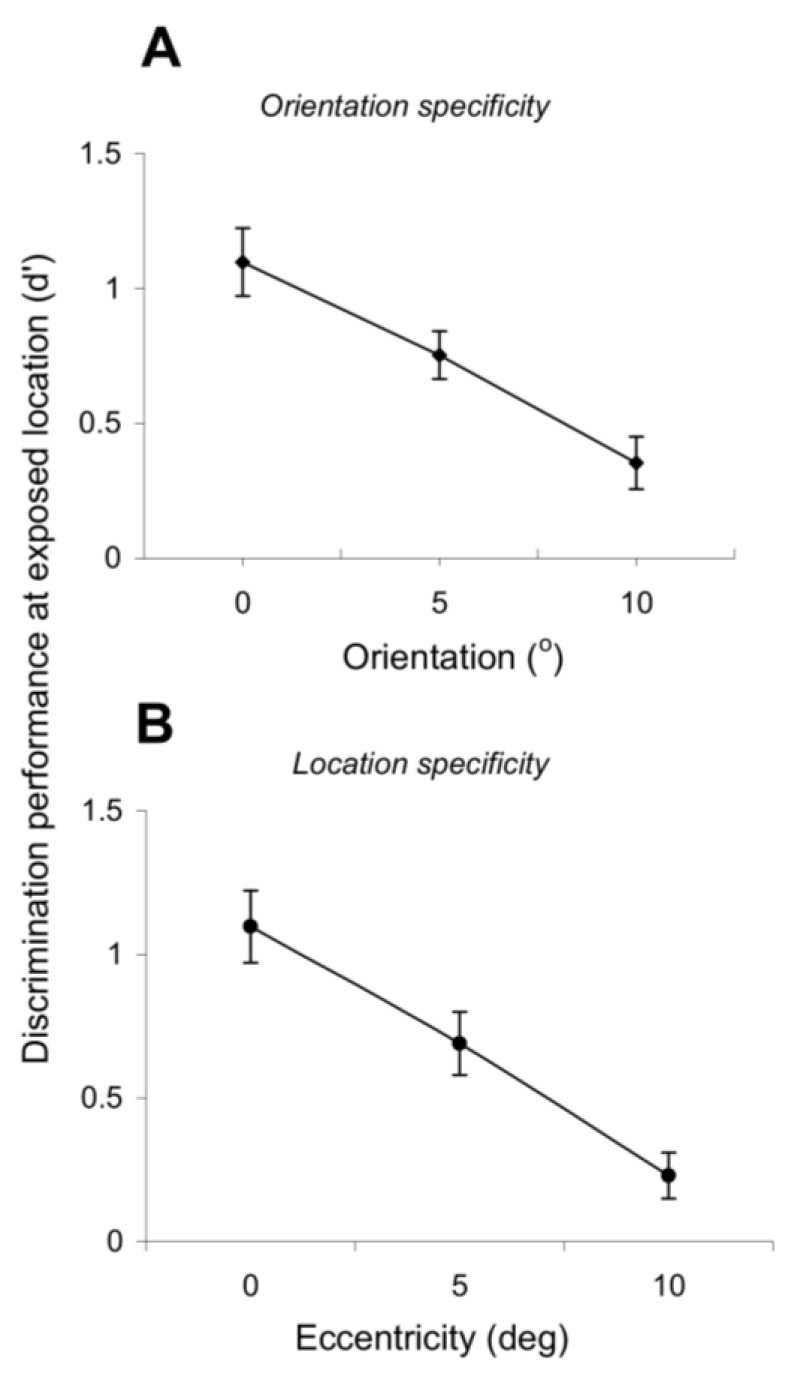
Specificity for orientation and retinal position. (**A**) Orientation specificity. The orientation discrimination performance at the exposed location gradually decreases as the test differs from the experienced orientations. Each point represents the mean performance (measured using d’) obtained by averaging data across four test sessions when the target orientation was 5° and 10° away from the exposed orientations (exposure to 60°–150° followed by tests at 65° and 70°). (**B**) Position specificity. The orientation discrimination performance at the exposed location gradually decreases as the eccentricity at which the orientation discrimination test was performed increases. Each point represents the mean performance (measured using d’) obtained by averaging data across four test sessions when the test location was 5 and 10 ° away from the exposed location (exposure to 0 ° eccentricity followed by tests at 5 and 10 ° eccentricity on the horizontal axis). Error bars represent SEM.

We further examined the degree of specificity of exposure learning by testing whether it depends on retinal position and orientation. Such dependency is a distinct feature of the perceptual learning of elementary features [9,10,13,14,31,32] and argues for the involvement of early and mid-level visual cortical areas in mediating perceptual learning as these areas are known to represent stimulus features with the finest resolution. Figure 4A,B reveals a high degree of orientation and position specificity of exposure learning. Indeed, the orientation discrimination performance at the exposed location (d’) gradually decreases as a function of increasing difference between the test and exposed orientations (Figure 4A; *p* < 0.01, Student’s *t*-test). Furthermore, the exposure-induced learning effects are spatially localized—Figure 4B shows that the orientation discrimination performance decreases as the eccentricity at which the orientation discrimination is performed increases (*p* < 0.01, Student’s *t*-test). Thus, exposure learning retains the position and orientation specificity of task-related practice learning.

**Figure 5:**
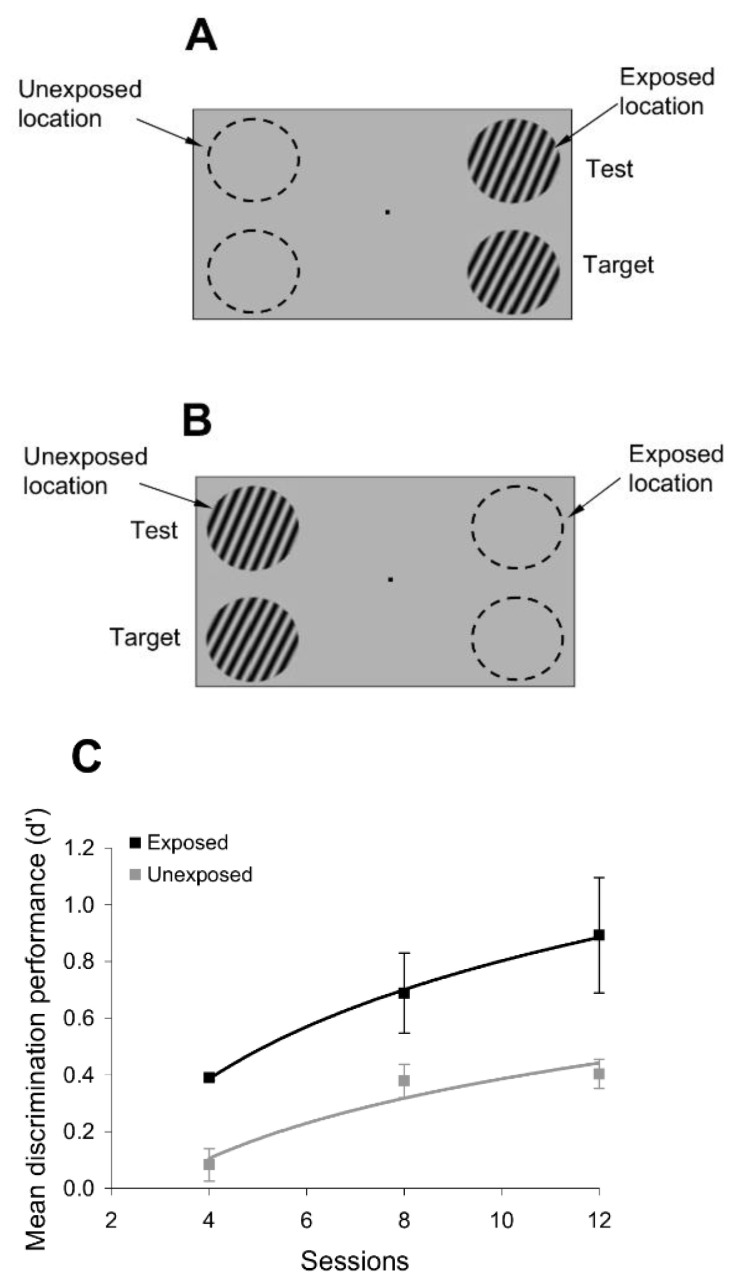
Simultaneous orientation discrimination. (**A**,**B**) Orientation discrimination performance was measured at both the exposed (**A**) and unexposed (**B**) locations by simultaneously presenting the test and target stimuli. The test was always presented at a fixed location (above the unexposed location). The center-to-center distance between the target and test locations was 5°. Each discrimination test was conducted after an exposure session similar to that discussed in Figure 1. (**C**) Exposure to orthogonal orientations significantly alters simultaneous orientation discrimination performance at the exposed location.

In principle, it could be argued that subjects may use orientation-insensitive mechanisms to distinguish between the target and test stimuli. Indeed, the timing of the stimuli in the test phase (200 ms) would be able to produce apparent motion. Hence, stimulus discriminations might be more easily performed by assessing the motion between the target and test rather than by comparing their orientation. Therefore, it is possible that the stimuli during the exposure stage may have altered the strength of the motion stimuli used in assessing the difference between the target and the test during the testing phase. To control for this possibility, we conducted exposure sessions similar to those described in Figure 1, but, after each exposure session, presented the test and target stimuli simultaneously during the testing phase (Figure 5A,B).

In these parallel orientation discrimination experiments, the test stimulus (of random orientation) was always presented at the exposed location, whereas the target stimulus was presented at a new, previously unexposed, location. If subjects rely exclusively on motion signals to distinguish between the target and the test, and if exposure alters the strength of those motion stimuli used in orientation discrimination, presenting the target and test stimuli in parallel should abolish the exposure-induced improvement in orientation discrimination performance. However, contrary to this prediction, Figure 5C shows that exposure to orthogonal orientations improves orientation discrimination performance in a significant manner (*p* < 0.05, Student’s *t*-test), even when the target and test stimuli are presented together. This demonstrates that exposure to orientation sequences induces effects that rely primarily on orientation, not motion, mechanisms.

It is also possible that the large difference between the orientation discrimination performance at the exposed and unexposed locations simply reflects a lack of stimulation at the unexposed location rather than an effect of exposure to orthogonal orientation sequences at the exposed location. To control for this possibility, we repeatedly stimulated a new location using a mask composed of a superposition of eight orientations equally spanning 0–180° that was flashed for 200 ms and was followed by a 200 ms blank (the stimulation duration was 3 min). However, although the mask was repeatedly presented for several sessions, we found that the orientation discrimination performance measured after exposure to these control stimuli was not significantly different from that at the unexposed location (*p* > 0.05, Student’s *t*-test).

### 3.2. The Relationship between Exposure and Practice Learning

We further examined whether passive stimulus exposure induces perceptual learning effects that are comparable to those induced by task repetition. Examining this issue is important as it has the potential to provide concurrent information about different types of perceptual plasticity that could be used to evaluate the effectiveness of bottom-up and top-down mechanisms. We devised a set of experiments to examine (i) the difference between the time course (strength and speed) of orientation-specific exposure and practice learning, and (ii) the degree of stimulus generalization of the effects induced by exposure and practice learning (using complex stimuli, such as shapes and natural scenes).

### 3.3. Time Course of Learning Effects

**Figure 6:**
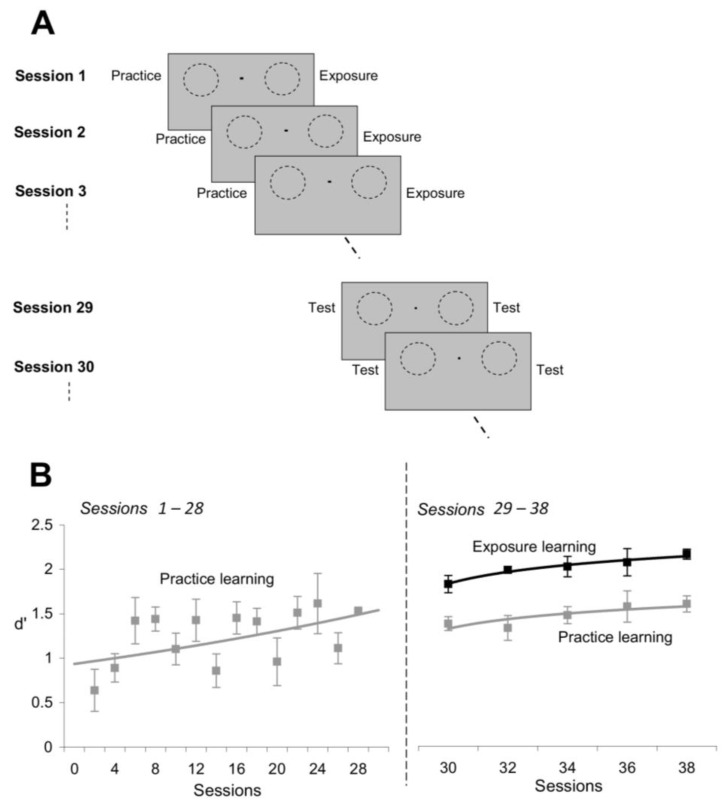
Exposure learning develops more rapidly than practice learning. (**A**) Experimental design. Subjects were required to experience practice learning at one location (left visual field) and exposure learning at another location (right visual field) for the same number of sessions (sessions 1 through 28). In sessions 29–38, orientation discrimination performance was measured at both exposed and unexposed locations to directly compare the effects of practice and exposure learning. (**B**) Exposure learning was stronger and developed more rapidly than practice learning. Black line: discrimination performance (d’) at the exposed location (exposure learning); gray line: discrimination performance at the unexposed location (practice learning). Error bars represent SEM.

We asked subjects to practice orientation discriminations at one location and, in a separate session, perform an exposure session in conjunction with a letter identification task at another location (Figure 6A). The two locations were symmetric with respect to the fixation point, and were stimulated in each session with the same number of stimuli, either nearby orientations (at the practice location) or orthogonal orientations (at the exposed location). The stimulus sequence for both the orientation discrimination practice sessions and the orientation exposure sessions was identical to the stimulus sequence from the experiments described in Figure 1. These two sets of stimuli were chosen in order to maximize performance for practice learning and exposure learning. Indeed, the most effective stimuli for practice learning are the stimuli of nearby orientation, whereas the most effective stimuli for exposure learning are the stimuli of orthogonal orientation (e.g., Figure 3D).

In agreement with previous perceptual learning studies [10,14,20], we confirmed that subjects gradually improved their orientation discrimination performance at the practice location during the time course of training (Figure 6B, left). Indeed, the mean orientation discrimination performance, d’, at the end of training (Sessions 25–28) was significantly higher than the mean d’ at the beginning of training (Sessions 1–4; *p* < 0.03, Student’s *t*-test) in all the subjects. However, contrary to common belief that practice learning is stronger than exposure learning, we found that repetitive exposure to orthogonal orientations led to superior learning (Figure 6B, right). That is, despite the fact that subjects did not perform any visual task at the exposed location for 28 sessions, they were nonetheless able to discriminate finer orientation differences at the exposed location compared to the practice location (Sessions 29–38). This demonstrates that passive exposure to orthogonal orientations induces stronger learning than task repetition (*p* < 0.008, Student’s *t*-test), and that exposure learning develops at a faster time course than practice learning.

### 3.4. Stimulus Generalization

Generalization is a key property of learning and is defined as a transfer of the improvement in perceptual performance achieved through training to new stimuli. Classical perceptual learning theories postulate the specificity of learning for the simple stimuli present during training, such as stimulus position and orientation, with little or no generalization to more complex stimuli. Indeed, there has been little evidence that practice learning leads to improvement in discrimination performance beyond the local stimulus configuration during task practice [9,13,15,18,19,20,32]. Given the importance of the generalization issue, we examined whether the improvement in orientation discrimination performance at the exposed location leads to improvement in the discrimination of complex shapes composed of orientation signals around the experienced axes, and then compared the degree of transfer of exposure and practice learning. Based on the rapid rate of acquisition of exposure learning (Figure 5), we expected that the discrimination of complex stimuli, such as shapes and natural scenes, would be improved more after repetitive exposure to orthogonal orientations, than after repetitive practice of orientation discriminations.

**Figure 7:**
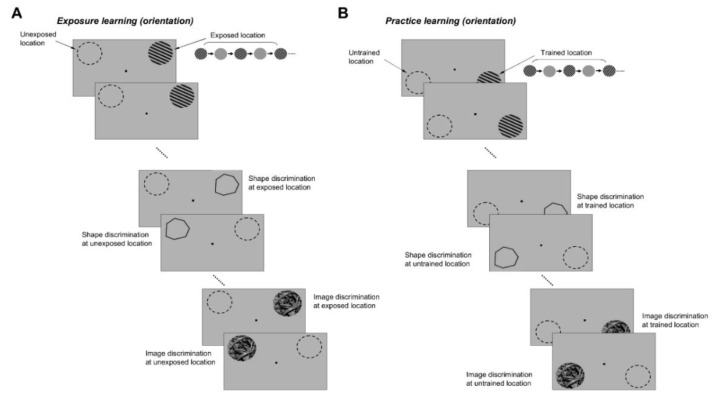
Stimulus generalization experiments. (**A**) Exposure learning. Subjects were successively exposed to two pairs of orthogonal orientations, 60°/150° and 30°/120°, 12 sessions for each pair. After completion of the orientation exposure sessions, subjects were required to discriminate shapes and natural scenes. Discrimination tests were performed at both exposed and unexposed locations by successively flashing shapes or natural images that were slightly tilted relative to each other. (**B**) Practice learning. At another spatial location, subjects practiced orientation discriminations around the four orientations used in the exposure experiment (12 sessions per orientation). After the completion of the orientation discrimination practice sessions, subjects were required to discriminate shapes and natural scenes at both the trained and untrained locations.

We tested the degree of stimulus generalization of exposure and practice learning by asking subjects to perform shape and image discrimination tasks after they had been exposed to orthogonal orientations and had practiced orientation discriminations for the same number of sessions (Figure 7). Thus, we conducted daily orientation exposure sessions similar to those described in Figure 1, in which subjects were exposed to two pairs of orthogonal orientations: 60°/150° and 30°/120° (in separate sessions; 12 sessions for each pair of orientations—Figure 7A). In pilot experiments (Suppl. Figure S2) we confirmed that exposure to two different pairs of orthogonal stimuli improved orientation discrimination along the four experienced axes (*p* < 0.03, Student’s *t*-test). The improvement in performance was restricted to the exposed location and did not impair or enhance performance around orientations other than the exposed ones. At another spatial location, we asked subjects to practice orientation discriminations around the four orientations used in the exposure experiment (12 sessions per orientation, Figure 7B). After the orientation exposure and discrimination practice sessions were completed, subjects were required to discriminate shapes and natural scenes.

These discrimination tests were performed at both pairs of exposed/unexposed and trained/untrained locations (Figure 7A,B) by successively flashing shapes or natural images that were slightly tilted relative to each other (a target stimulus was followed by a test stimulus that was either identical to the target or was rotated by ±2°, ±3°, ±4°, and ±5° with respect to the target). At the end of each trial, subjects were asked to judge whether the test was similar or different from the target (the number of match and non-match stimuli was identical). We reasoned that since complex stimuli have a rich representation in the orientation domain [23,33,34], improving orientation discrimination through repetitive exposure or practice could lead to an improvement in the discrimination of those complex stimuli with strong orientation signals around the exposed or trained axes. We controlled the orientation content of the complex stimuli by generating polygons with four of the sides oriented along the exposed axes (Figure 2A), and natural scenes of broad orientation spectrum, including orientation signals of various strengths along the experienced axes (Figure 2B). We evaluated the orientation content of each image by calculating the orientation magnitude histogram, and, for each histogram, computed an exposure orientation index (EOI, see Section 2) that quantifies the relative strength of orientation signals along the experienced axes.

**Figure 8:**
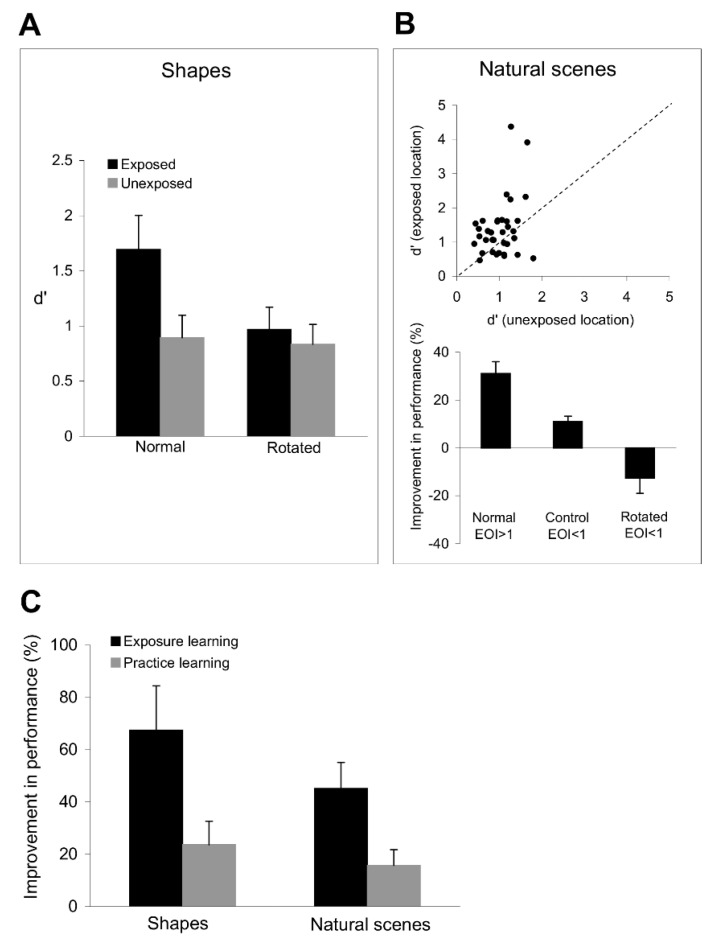
Results of the stimulus generalization experiments. (**A**) Shape discrimination performance is improved after exposure to orthogonal orientation sequences. In the ‘normal’ condition, i.e., shapes were not rotated, there is a significant improvement in shape discrimination performance only at the exposed location (black bars), whereas there is no improvement in performance at the unexposed location (grey bars). In the ‘rotated’ condition, we rotated the target and test polygons by 20°, in which case the improvement in shape discrimination performance at the exposed location was abolished. (**B**) Top: Image discrimination performance is improved after exposure to orthogonal orientation sequences. The scatter plot represents the discrimination performance at the exposed location vs. that at the unexposed location. Each point represents the d’ value for one subject and one image (averaged across 3 sessions). Only images with an exposure orientation index greater than 1 were included in the analysis. Bottom: Exposure to orthogonal orientations does not improve the discrimination of images with weak orientation signals along exposed axes (EOI < 1) and the discrimination of images that were rotated by 20°. We rotated only the images that had strong orientation signals along the exposed axes (EOI > 1 before rotation). (**C**) Mean improvement in shape and image discrimination performance at the exposed and trained locations (relative to the unexposed and untrained locations). Subjects exhibited a larger mean improvement in shape and image discrimination performance after exposure learning (black bars) than after practice learning (grey bars). The improvement in discrimination performance was compared in 4 sessions at the exposed and trained locations in each subject. Error bars represent SEM.

The results in Figure 8A,B demonstrate that exposure to orthogonal orientations leads to a robust improvement in the discrimination of complex visual stimuli despite the fact that subjects had no prior experience with these stimuli. Indeed, Figure 8A shows a significant improvement in shape discrimination performance at the exposed location (*p* < 0.03, Student’s *t*-test), likely reflecting an increase in neuronal orientation discrimination performance around the experienced axes after repeated exposure. To test whether there is a direct relationship between the improvement in orientation discrimination around the experienced axes and the shape discrimination performance, we conducted control experiments in which we rotated the shapes by 20°, such that all the sides of the polygons were now presented at orientations that differed by more than 10° from the experienced ones. Since orientation exposure improves orientation discrimination performance only around the experienced orientations (Figure 3), we found that rotating the target and test shapes abolished the improvement in shape discrimination performance (Figure 8A), which was similar at the exposed and unexposed locations (*p* > 0.1, Student’s *t*-test).

Importantly, exposure to orthogonal orientation sequences leads to a significant improvement not only in shape discrimination performance, but also in the ability of the visual system to discriminate natural images. Figure 8B (top) shows that the image discrimination performance at the exposed location is significantly better than that at the unexposed location (*p* < 0.01, Student’s *t*-test). We controlled the orientation content of each image by dividing the images in our set into two groups depending on whether their exposure orientation index, EOI, was greater or smaller than 1. Figure 8B (bottom) shows that for those images with strong orientation components along the experienced axes (EOI > 1), subjects were more likely to indicate that the test and target images were different at the exposed location than when they were presented at the unexposed location (*p* < 0.03, Student’s *t*-test). Importantly, exposure to orthogonal orientations did not improve discrimination performance (*p* > 0.5, Student’s *t*-test) for images with weak orientation signals (EOI < 1) along the experienced axes (Figure 8B, bottom), possibly due to lack of exposure-induced plasticity in neurons tuned to those orientations. We further examined the link between the exposure-induced improvement in orientation and image discrimination performance by rotating the images with strong orientation signals along exposed axes by 20° (this procedure dropped EOI to values < 1 in each image) followed by image discrimination. Despite the fact that the images before rotation were associated with a significant improvement in discrimination performance at the exposed location, weakening the correlation between the orientation content of the image and the experienced orientations (by rotation) abolished the original improvement in discrimination performance (*p* > 0.1, Student’s *t*-test).

These data indicate that the improvement in the discrimination of simple stimuli after exposure generalizes to complex stimuli that include combinations of elementary stimuli. This raises the issue of whether practice learning exhibits a similar degree of stimulus generalization. We directly compared the degree of specificity of bottom-up and top-down plasticity by examining the subjects’ capacity to discriminate complex shapes after they completed 12 successive sessions of exposure and practice learning. Figure 8C illustrates the mean improvement in shape and image discrimination performance at the exposed and trained locations (relative to the unexposed and untrained locations). Confirming our prediction, subjects exhibited a larger improvement in shape (*p* < 0.01, Student’s *t*-test) and image (*p* < 0.03, Student’s *t*-test) discrimination performance after exposure learning than after practice learning (comparing the improvement in discrimination performance in four sessions at the exposed and trained locations in each subject).

## 4. Discussion

Our results indicate that the underlying principles of adaptive learning should be revised. Although it is well accepted that ‘practice makes perfect’, we show here that repeated practice of a specific task may not always be the most efficient way to improve behavioral performance. Practice learning and exposure learning have long been considered as the major forms of perceptual plasticity in the adult brain, and yet no study to date has investigated them concurrently. Our study shows that repetitive exposure to unattended oriented stimuli induces a form of plasticity that develops at a higher rate and exhibits greater stimulus generalization than practice learning.

The issue of whether sensory systems have the capacity to adapt as a result of passive stimulus exposure has rarely been examined. In one of the first studies of perceptual effects of stimulus exposure [27], it was reported that a subliminal presentation of a neutral stimulus can bias subsequent preference judgments (see also [26]). More recently, repetitive exposure to a background motion signal below the visibility threshold has been shown to improve performance specifically for the direction of the exposed motion during subsequent suprathreshold motion tests [28]. In the orientation domain, it has been previously shown that irrelevant stimuli outside the spotlight of attention are not ignored by the visual system, but are in fact processed [35]. However, whether passive exposure to orientation stimuli can induce learning has been unknown. We demonstrate here that exposure learning can operate in the orientation domain. In contrast to the fact that exposure to weak motion stimuli improves subsequent motion discrimination along the exposed direction, we found that repetitive exposure to a fixed orientation induced only a mild (statistically nonsignificant) improvement in the discrimination of nearby or orthogonal orientations (cf. Figure 2A,B). However, importantly, we found that repetitive exposure to orthogonal, but not similar or nearby orientations, caused a strong and persistent improvement in orientation discrimination performance. These differences between exposure learning in the orientation and motion domain may possibly reflect the different neuronal substrate for motion and orientation processing.

What type of mechanism could underlie the exposure-induced improvement in orientation and complex shape discrimination performance demonstrated here? Our results argue for the involvement of feature-specific bottom-up mechanisms that are initiated in the early visual cortex, and, subsequently, affect neuronal performance along the entire visual pathway. Indeed, exposing visual cortical neurons to an orientation that is orthogonal to their preferred has been shown to transiently sharpen individual orientation tuning curves [23,25], whereas exposure to similar or nearby orientations has been shown to broaden orientation selectivity [23,24]. The changes in neuronal selectivity after brief stimulus exposure have been explained by invoking a combination of short-term synaptic plasticity mechanisms [36,37] and intracortical interactions [23,24]. Therefore, it is conceivable that repeated exposure to orthogonal orientations could induce long-term plasticity in the cortical local circuits activated by the exposed stimuli, which would sharpen orientation selectivity to improve neuronal discrimination performance along the exposed axes. In contrast, repeated exposure to similar or nearby orientations would induce only mild changes in neuronal performance around the exposed orientations. Subsequently, we propose that neurons at the later stages of visual processing could pool and differentiate the signals from early areas to amplify neuronal discrimination performance when complex shapes are presented.

Nonetheless, although cortical mechanisms have a proven role in the mechanism that underlies the kind of perceptual behavior we are describing here, the contribution of subcortical structures to bottom-up plasticity should also be taken into consideration. Duménieu et al. show in their review that the role of dorsal lateral geniculate nucleus (dLGN) neurons extends to complex integration of visual signals arising from the retina, the cortex, and the superior colliculus (SC) [38]. Furthermore, dLGN neurons also participate in cognitive functions and express functional and synaptic plasticity. Similarly, SC neurons also display cognitive functions, express functional and synaptic plasticity, and have recently been shown to be involved in working memory and in saccade adaptation. Consequently, it is reasonable to at least consider these subcortical structures as contributors to adaptive learning in addition to perception learning.

The difference in speed and generalization between exposure and practice learning could possibly reflect the difference in the magnitude of the effects induced by single exposure and discrimination practice trials at the neuronal level. Thus, although practicing orientation discrimination improves the ability of the visual system to identify finer orientation differences after each training session, the associated changes in neuronal responses and sensitivity are typically small. Indeed, recent studies in the monkey visual cortex have failed to find large changes in the response properties of individual visual cortical neurons after sessions of practice learning. Intensive practice in discriminating small orientation differences did not lead to significant changes in neuronal responsiveness or selectivity in V1 (e.g., [20,39], who reported only subtle changes in the slope of orientation tuning curves after learning). In area V4, training in an orientation discrimination task led to stronger responses and narrower orientation tuning curves [40], but these changes were subtle and were observed only after extended practice. In contrast, passive exposure or adaptation to a stimulus of fixed orientation has been shown to induce more robust changes in neuronal responses and sensitivity [23,24]. This raises the possibility that repeated exposure to orientation sequences could rapidly trigger bottom-up plasticity in early and mid-level visual cortical areas, whereas practice learning could induce slower forms of top-down plasticity that could begin in mid-level, but not early, cortical areas to account for the differences in the time course of exposure and practice learning.

Our result that exposure learning generalizes to a larger extent than practice learning is consistent with recent studies investigating the relationship between the difficulty of the learning task and the degree of stimulus generalization. Indeed, although attention-based practice learning has been traditionally believed to show little generalization, more recent evidence showed that the degree of learning specificity may depend on task difficulty [18]. Thus, as task difficulty is decreased, learning becomes less specific with respect to simple stimulus features, such as orientation and retinal position, and thus learning exhibits a higher degree of generalization. Since exposure learning involves only passive stimulus presentation (there is no task at the exposed location—easy condition), whereas practice learning involves a difficult task that allocates significant attentional resources, it could also be argued that the difference in the degree of generalization between exposure and practice learning could be due to the differences in the difficulty of the two tasks.

## 5. Conclusions

Altogether, our results demonstrate the flexibility and capacity for plasticity of visual system when it is repetitively exposed to irrelevant incoming stimuli. Thus, despite the fact that subjects had similar experience with the stimuli presented during exposure and practice learning, the rate and stimulus generalization of exposure learning were greater than those of practice learning. We suggest that long-term sensory adaptation by passive exposure constitutes a primitive form of plasticity that is complementary to practice, attention-based learning. Indeed, it is commonly assumed that optimal performance levels are achieved only if attention is restricted to a small fraction of the environmental signals, in a way that prevents irrelevant stimuli from being processed. However, given the richness of the information that is not attended, it makes biological sense that the brain has developed mechanisms to take advantage of the signals outside the spotlight of attention. It is thus possible that unattended signals could have the capacity to induce strong bottom-up plasticity and cause learning on the basis of their frequency of occurrence. Our results indicate that exposure learning may offer certain advantages by limiting the constraints of practice learning on speed and generalization. Although it may appear that the main disadvantage of exposure learning is that it allows irrelevant information to be processed by sensory systems and influence behavior, the effects induced by passive exposure may contribute to directing attentional resources toward previously ignored, but frequently presented, environmental stimuli.

## Data Availability

Not applicable.

## Supplementary Materials

The following supporting information can be downloaded at: https://www.mdpi.com/article/10.3390/brainsci12040508/s1, Figure S1: Preliminary experiments; Figure S2: Exposure to two pairs of orthogonal orientations.
