# Correction: Iliescu, B.F. Learning by Exposure in the Visual System. *Brain Sci.* 2022, *12*, 508

**DOI:** 10.3390/brainsci12111528

**Published:** 2022-11-11

**Authors:** Bogdan F. Iliescu

**Affiliations:** Neurosurgery Department, Gr T Popa University of Medicine and Pharmacy, 700115 Iasi, Romania; bogdan-florin.iliescu@umfiasi.ro

## Incorrect Authorship

Bryan Hansen and Valentin Dragoi were previously included as authors in the publication [1] improperly without their permission. According to the ICMJE guidelines, all of the authors agreed to delete Bryan Hansen and Valentin Dragoi from the authorship.

The correct author is “Bogdan F. Iliescu”.

Affiliation should be changed to “Neurosurgery Department, Gr T Popa University of Medicine and Pharmacy, 700115 Iasi, Romania”.

The author state that the scientific conclusions are unaffected. The original publication has also been updated.
