# Supplementary material for: Learning by Exposure in the Visual System
Source: Brain Sci. 2022 Apr 17;12(4):508. doi: 10.3390/brainsci12040508 (PMC9027739; doi:10.3390/brainsci12040508)
Supplement: Supplementary file 1 [file brainsci-12-00508-s001.zip › brainsci-1651077-supplementary.pdf]

Controlling attention during exposure

During the exposure stage, attention was controlled by asking subjects to complete a letter identification task (presented above the fixation point, Figure S1A), in which a new letter was randomly presented every 200 ms. The location of the letter identification task was chosen on the basis of pilot tests conducted prior to the exposure experiments to ensure that the exposed and unexposed locations were unattended when subjects performed the letter task at a performance level of 80% or more. To measure the extent to which subjects attended the exposed location during the letter identification task, we asked subjects to fixate in the center of the screen while oriented sine-wave gratings were repeatedly flashed for 200 ms each and were separated by a 200 ms blank interval (Figure S1A; the grating and letter stimuli were simultaneously turned on and off). The stimulus sequence was composed of 450 gratings that were repeatedly presented for 3 min.

The gratings had the same orientation, except for a small percentage (< 10%) that had an 'odd', orthogonal, orientation. The number of odd stimuli ranged between 27 and 41, and was randomly varied each session (the interval between two consecutive odd stimuli was greater than 1 s). Throughout the pilot experiments, subjects were required to count how many times a specific letter was present in the sequence (at the letter location), and, simultaneously, press a key within 1 s since an odd stimulus was detected at the exposed location. Suppl. Figure S1B illustrates the results of the pilot experiments obtained in two subjects during 10 sessions when the location of the letter task was 7 deg above the fixation spot. These results were obtained when the performance in the letter identification task was higher than 80%, by plotting the number of key presses (or 'hits') as a function of the number of odd gratings. Since the mean performance in the orientation detection task (at the exposed location) was <10%, which was lower than the detection performance in catch trials (the number of odd gratings was 0), we concluded that the exposed location was unattended.

The orientation discrimination performance, d′, was calculated for each orientation difference using the equal-variance model. d′ is the standardized difference between the means of the Signal Present and Signal Absent distributions. To calculate d′, we need to know only a person's hit rate and false alarm rate. The formula for d′ is as follows: $d' = z(FA) - z(H)$, where FA and H are the False Alarm and Hit rates, respectively, that correspond to right-tail probabilities on the normal distribution. Thus, z(FA) and z(H) are the z-scores that correspond to these right-tail p-values represented by FA and H. Larger absolute values of d′ mean that a person is more sensitive to the difference between the Signal Present and Signal Absent distributions. d′ values near zero indicate chance performance.

Exposure to two pairs of orthogonal orientations

The results in Figure S2 show that passive exposure to orthogonal orientation sequences improves orientation discrimination along the exposure axes. This raises the issue of whether exposure to two different pairs of orthogonal stimuli improved orientation discrimination along the four exposed axes. To test this possibility, we conducted daily orientation exposure sessions similar to those described in Figure 1, in which subjects were exposed to two pairs of orthogonal orientations: 60o/150o and 30o/120o (in separate sessions; 12 sessions for each pair of orientations). Suppl. Figure S2 shows that the exposure to two different pairs of orthogonal stimuli improved orientation discrimination along the four experienced axes ($P < 0.03$, Student's t-test). The improvement in performance was restricted to the exposed location and did not impair or enhance performance around orientations others than the exposed ones.

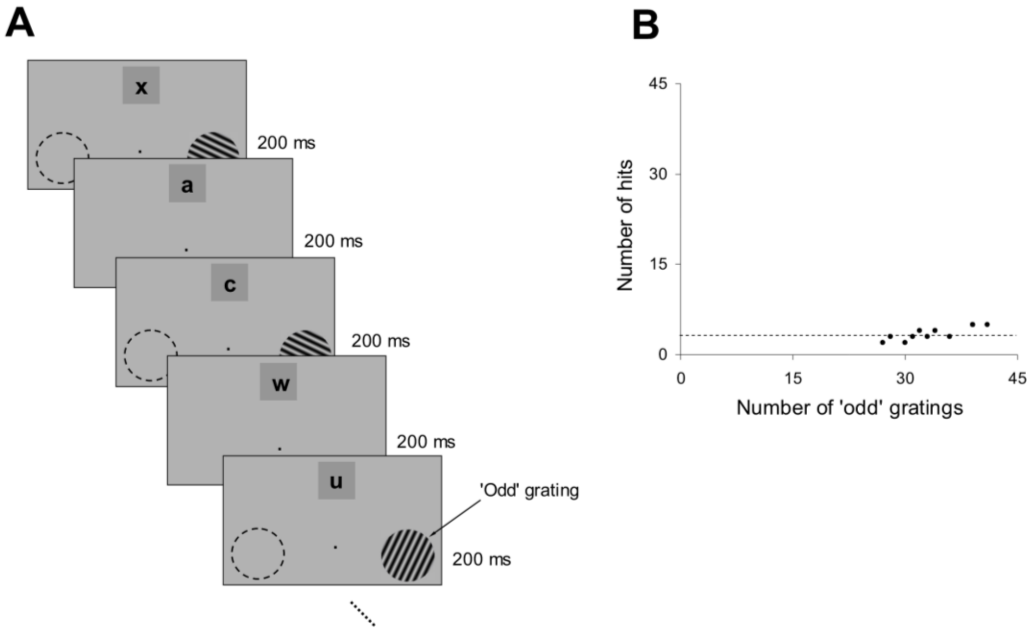

Figure S1. Preliminary experiments to assess whether the exposed and unexposed locations were unattended when subjects performed the letter identification task. (**A**) Experimental design. Subjects were required to count how many times a specific letter was present in the sequence and, simultaneously, press a key within 1 s since an odd stimulus was detected at the exposed location. (**B**) The graph represents the number of key presses (or 'hits') as a function of the number of odd gratings in one session. The results of the pilot experiments indicate that the exposed location was unattended (detection performance was similar to that in the catch trials).

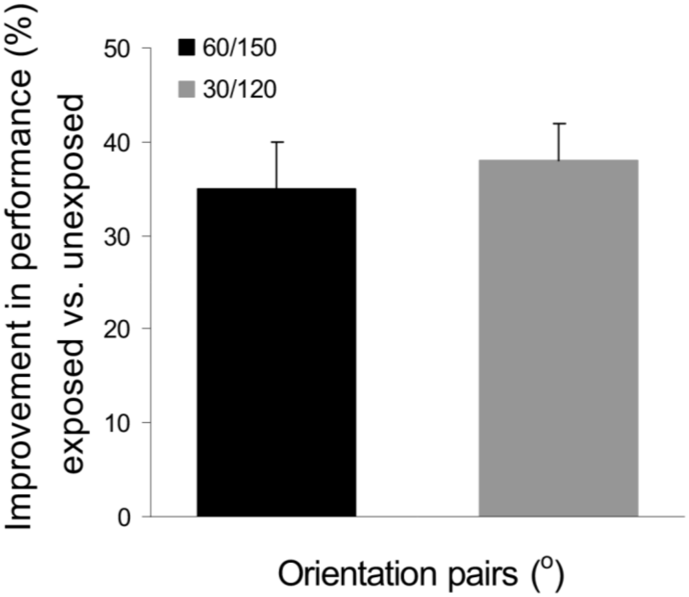

Figure S2. Exposure to two pairs of orthogonal orientations. Subjects were exposed to two different pairs of orthogonal orientation sequences (60o/150o and 30o/120o), and this exposure led to an improvement in orientation discrimination (exposed vs. unexposed locations) along the four experienced axes (mean of six subjects). Black bar: improvement in orientation discrimination performance after exposure to the

60o/150o sequence; gray bar: improvement in orientation discrimination performance after exposure to the 30o/120o sequence. Error bars represent s.e.m.
